# COVID-19, the European Health Union and the CJEU: Lessons from the Case Law on the Banking Union

**DOI:** 10.1017/err.2020.78

**Published:** 2020-09-01

**Authors:** Oliver BARTLETT

**Affiliations:** * Maynooth University Law Department, Maynooth, Co Kildare, Ireland; email: ollie.bartlett@mu.ie.

## Abstract

This contribution will draw on the literature that has accumulated on how the Court of Justice of the European Union (CJEU) has responded to the European Banking Union, which was established in similar crisis circumstances that now face the European Union (EU) in the age of COVID-19, to illustrate the legal issues on which the CJEU’s input will be particularly important in the shaping of any future European Health Union. This contribution will highlight three legal issues in particular: the interpretation of EU Treaty provisions in the health field; the scope of EU competence; and the powers of EU institutions and agencies. This contribution will argue that the CJEU case law to date on health issues is encouraging with respect to competence and agencies, but more cautious with respect to the Treaty’s health provisions.

## I. Introduction

The Court of Justice of the European Union (CJEU) has given a number of pivotal judgments in cases that have dealt with the intersection of health policy and EU law, and in so doing has played a key role in the emergence of EU health law as a distinct field. EU health law has developed to a point that scholars have called for the creation of a distinct European Health Union to consolidate common European rules and standards that will contribute to improving the health of all Europeans.^1^ As the implications of the COVID-19 pandemic for European cooperation on health issues become clearer, the calls for a European Health Union have been reinforced.^2^ In light of such a seismic shift in how the world perceives the health, economic and social risks inherent in an uncontrolled global infectious disease outbreak, there will probably never be a better stimulus for EU Member States to develop the tools for enhanced formal cooperation on health issues.

It is clear that if a Health Union is created, the CJEU would play an important role in its development and in whether it is eventually successful in improving Europe’s preparedness and resilience against future infectious disease crises. The CJEU has played this role before. In the aftermath of the Eurozone crisis from 2008 to 2010, EU Member States responded with the formation of a Banking Union, and the CJEU was called upon to interpret the compatibility of its various parts with EU law. The CJEU’s decisions enabled the Member States to create stability in the financial sector and to safeguard against similar crises developing in the future.

This contribution will draw on the literature that has accumulated on how the CJEU has responded to the Banking Union, which was established in similar crisis circumstances that now face the EU in the age of COVID-19, to illustrate the legal issues on which the CJEU’s input will be particularly important in the shaping of any future Health Union. There are three legal issues that this contribution will highlight: the interpretation of Treaty provisions in the health field; the scope of EU competence; and the powers of EU institutions and agencies. This contribution will examine each of these in turn and will argue that the CJEU case law to date on health issues is encouraging with respect to competence and agencies, but more cautious with respect to the Treaty’s health provisions.

## II. Learning from the CJEU’s response to the Banking Union

Between 2008 and 2010, a sovereign debt crisis developed in Europe. Several countries, under the weight of the preceding financial crisis, became at risk of defaulting on their national debts, which created the subsequent risk that the Eurozone itself might collapse. To prevent this from occurring, EU Member States put in place a series of measures with the ultimate aim of creating a Banking Union – a single governance structure for the Eurozone’s financial institutions, the objective of which was to ensure the responsible management of banking and financial activity and, perhaps even more importantly, to establish a response mechanism for dealing effectively with any financial institution that did find itself in trouble. Two Regulations establishing the Single Supervisory Mechanism (for monitoring European financial institutions)^3^ and the Single Resolution Mechanism (for responding to problems in Europe’s financial institutions)^4^ constitute the current legal core of the Banking Union.

The Eurozone crisis and the current public health crisis are similar in the risk posed to economic and social stability and in the governance challenges facing EU Member States. The key lesson seemingly learnt in the aftermath of the global financial crisis and European sovereign debt crisis was that, given the interconnectedness of global financial networks, a failing institution in one country could spread devastation to other countries. Thus, the essential question became how, in a policy area that had always been politically sensitive, should EU Member States use European institutions to build the level of cooperation required to ensure that a crisis that could not be contained by national law did not happen again. This is exactly the same question now facing the Member States in the ongoing COVID-19 crisis – how should EU Member States use European institutions to build the unprecedented level of cooperation in the health field that will be essential if Europe’s healthcare systems are to continue managing COVID-19 and overcome future infectious disease outbreaks?

This contribution contends that it is possible to identify certain key areas of CJEU jurisprudence that have been crucial in determining the EU Member States’ success in building the Banking Union and to identify how the CJEU might react on those same issues in the context of a future Health Union. It is argued – based on the experience of the Banking Union – that the CJEU will have to respond in similar ways if a Health Union is to be successfully constructed to effectively respond to the COVID-19 crisis. The sections below will briefly explore the three areas identified in Section I,^5^ addressing first the Banking Union case law and then the case law on health.

## III. Treaty provisions in the health field

It is clear that the successful development of a Health Union will depend upon whether the CJEU is prepared to interpret the health-related provisions of the EU Treaties boldly. The Banking Union has been able to develop more or less uninhibited due in part to the fact that the CJEU has made active efforts to interpret relevant Treaty provisions in a manner that politically supports deeper banking integration.

In *Pringle*,^6^ the CJEU was asked whether the establishment of the European Stability Mechanism (ESM), a permanent Eurozone fiscal backstop measure created by an international treaty independent from the EU legal order, was compatible with EU law. In addition to several substantive questions, the CJEU first had to address the arguments of ten Member States plus the European Commission and the European Council that it did not have the right to give judgment on these substantive issues. Clearly, the Member States were concerned that if the CJEU did not support the creation of the ESM, the plan to recover from the Eurozone crisis could be put in jeopardy. Thus, they argued that the CJEU’s right to review the establishment of the EMS was “limited, if not excluded, because the question relates to the validity of primary law”, and that “the Court has no power under Article 267 TFEU to assess the validity of provisions of the Treaties”.^7^ However, the CJEU rejected these arguments on the basis that it has responsibility under Article 19(1) TEU to ensure that the law is observed in the interpretation and application of the Treaties. Since the questions put to it related to how the Member States had used the simplified Treaty revision mechanism in Article 48(6) TEU to provide a legal basis for the ESM by amending Article 136 TFEU, as well as whether various provisions of the TFEU (in addition to the general principles of effective judicial protection and legal certainty) could preclude a Eurozone Member State from concluding and ratifying the ESM Treaty, it was the CJEU’s responsibility to answer those questions.^8^ This was a bold and progressive step, since it was “the first example, in the EU legal order, of the judicial control of the constitutionality of constitutional change”,^9^ and a politically sensitive one given that the future of European banking integration would hinge on the legitimacy of the Member States’ actions in adopting the ESM Treaty.^10^ The CJEU’s willingness to give novel – although some would argue strained – interpretations of relevant Treaty provisions was undoubtedly a result of its acceptance of the political realities involved,^11^ and this provided crucial support for the nascent Banking Union project.^12^

This attitude can also be observed in the *Rimšēvičs* ^13^ judgment, which concerned the prosecution of the Latvian Central Bank governor and an order by the Latvian authorities that he be removed from his post pending investigation. The order was challenged under Article 14(2) of the European Central Bank (ECB) Statute,^14^ which it appeared allowed the applicants to bring their case directly to the CJEU. Latvia argued in response that the CJEU had no power under Article 267 TFEU to review the validity of national law enforcement agencies. This was the first time that Article 14(2) had ever been invoked, but the CJEU held that it could hear the case, and moreover held that Article 14(2) did grant it the power to annul the Latvian authorities’ order, insisting that direct recourse to the CJEU was essential to safeguard the European function that national central bank governors perform. This was a bold interpretation of an untested constitutional provision, which served to prevent the effective operation of European-level banking supervision being undermined and was welcomed by commentators as a show of political support for the independence of the ECB Governing Council and thus the wider process of banking integration.^15^

If the CJEU is to protect a future Health Union in the way that it has protected the Banking Union, it will need to be bold in interpreting Treaty provisions that have yet to be tested in the context of deepening health integration. The CJEU’s existing case law on health gives us some reason for optimism in this regard, but also some reason for caution.

By now it is clear that the CJEU is comfortable with making unprecedented decisions in its internal market case law to Europeanise the provision of health care.^16^ Cases like *Luisi and Carbone* ^17^ and *Kholl* ^18^ showed that the CJEU was not afraid to bring sensitive decisions on health resource allocation within the scope of the freedom to provide services and create new health rights for Europeans.^19^ However, the CJEU has also shown some restraint on issues that are particularly politically sensitive. For example, in *Grogan*,^20^ the CJEU gave a cautious judgment that largely avoided subjecting the choices of the national legislature on abortion services to strict review, and in *Gourmet*,^21^ the CJEU also refused to take a position on the proportionality of national alcohol regulations, passing the decision instead to the national court. Thus, the CJEU is not single-minded when it comes to the application of EU law to politically sensitive health issues – there are clearly some health issues on which restraint is worth more to the CJEU than economic integration.

If we focus on how the CJEU has interpreted EU Treaty provisions in the health field – particularly Article 168 TFEU, which imposes an obligation upon the EU to consider how its policies in all fields could promote health – there are reasons to be more cautious about whether the CJEU would be bold in its interpretations in support of a future Health Union. Although the CJEU itself has not been asked directly about the nature of the obligation in Article 168, the General Court has had to tackle this question on a handful of occasions, and we can gain some insight from the General Court’s answers of how the CJEU might react if pushed to define the full extent of the powers granted by Article 168.

The General Court has appeared quite reticent to actually assess whether the substance of the Article 168 obligation is being fulfilled. In *TestBioTech*,^22^ the General Court accepted that the EU institutions are bound by Article 168, but it was unwilling to articulate what exactly constitutes an inadequate level of health protection under Article 168. In *Spain v Commission*,^23^ the General Court was content to rule that Spain had not put forward enough evidence to prove that the Commission had neglected its duty under Article 168 to secure a high level of health protection, but was not willing to consider in any detail what level of evidence would actually be sufficient.

Thus, in contrast to the CJEU’s boldness in interpreting Treaty provisions that are crucial to banking integration, the General Court does not appear to have been comparably bold in interpreting the obligations imposed by Article 168. Any conclusions on what this tells us about the CJEU’s willingness to politically defend and support a Health Union must of course be cautious. When interpreting primary law relevant to the Banking Union, the CJEU has diverged from the opinions of the other EU judicial institutions on notable issues,^24^ so the conservatism of the General Court noted above does not necessarily indicate that the CJEU would be equally conservative in interpreting Article 168, or any new health provisions that might be inserted into the Treaties in the future.

Having said this, there are instances where the CJEU has refused indirect opportunities to give an expansive interpretation of Article 168. For example, when asked whether the obligation to ensure a high level of health protection in all policies implied that regulations on tobacco product labelling should be applied to tobacco products designated for export to non-EU countries in the absence of clear legislative intent (on account of the mainstreaming of health concerns into the Common Commercial Policy), the CJEU was silent on the application of Article 168 and held that no such implication could be drawn.^25^ When asked whether Article 168 supports reduced rates of value-added tax (VAT) being applied to both medicinal products and medical devices, despite the EU’s common VAT rules indicating that only the former can benefit from reduced VAT rates, the CJEU held that Article 168 did not support such an interpretation because the “the objective pursued by that provision – to set high standards of quality and safety – differs substantially from that pursued by Annex III to Directive 2006/112”,^26^ even though the purpose of Article 168 is precisely to prompt consideration of how policy in other fields can expand health protections alongside whatever other objectives are sought. Thus, there is some evidence to suggest that the CJEU itself might well be cautious in offering radical interpretations of EU primary law relating specifically to health policy, which could potentially be problematic for a future Health Union. However, this cautiousness may be offset by its evident willingness to interpret other provisions of EU primary law, specifically those relating to the EU’s internal market competence, in a way that supports the health policy objectives of the Union.

## IV. Competence

It is clear from the Banking Union case law that establishing the legitimacy of the constitutional foundations of any future Health Union will be key, given the sensitivity of the issues at stake and the number of stakeholders that might benefit from a reduced EU role in health policy. The choice of competence for a Health Union is perhaps the easiest target for any party seeking to challenge its legitimacy, since there is no explicit competence within the Treaty to harmonise the rules of the Member States on health issues. This could become less of a problem if the Member States were to create such a competence, as is explored in other contributions to this Special Issue. However, given the unlikeliness of this, the robustness of the EU’s existing suite of competences for supporting a Health Union will likely come under scrutiny. This was exactly the case with the development of the Banking Union, and the CJEU played a key role in confirming that the use of the EU’s existing harmonisation competences to build a Banking Union was constitutionally legitimate.

In the *ESMA* ^27^ judgment, the UK challenged Article 28 of the Short Selling Regulation, which gave the European Securities and Markets Authority (ESMA) regulatory and enforcement powers over the practice of short selling – selling borrowed securities for full price and buying them back for a profit when their value drops – a practice that contributed to the financial and Eurozone crises and whose regulation by the Member States until the adoption of the Regulation had been fragmentary.^28^ In addition to questions on agency powers, the CJEU was asked whether Article 114 provided the competence to grant extensive regulatory and enforcement powers to the ESMA. The CJEU decided that it did. Although the judgment’s legal robustness has been questioned, this was perhaps inevitable given that the CJEU was clearly concerned to give a ruling that provided stability for the wider constitutional foundations of the Baking Union.^29^ After all, if the grant of banking supervisory powers to the ESMA was unconstitutional, then the very existence of the Banking Union itself, also founded upon Article 114, might be unconstitutional as well.^30^ Thus, the CJEU’s answer on competence reflected “considerable sensitivity to pressing practical and political realities”.^31^

This same sensitivity can be observed in the CJEU’s jurisprudence on the use of Article 114 TFEU to harmonise health regulations, giving us reason to believe that the CJEU would also support the use of the EU’s existing harmonisation powers to build a Health Union in the face of crisis. The *Tobacco Advertising* ^32^ judgments are the obvious candidates for evidence of this. Although it initially held in *Tobacco Advertising 1* that the competence granted by Article 114 could not be used to adopt a directive on tobacco advertising, when the CJEU was presented in *Tobacco Advertising 2* with a revised directive that fitted the (very flexible) conditions for use of Article 114 that it had laid out in *Tobacco Advertising 1*, it confirmed that the purpose to which Article 114 is put is irrelevant as long as the conditions are met. There have been several subsequent challenges to the legal basis of EU tobacco control legislation, all of which have been rejected.^33^ In this case law, the CJEU demonstrates an awareness of the complexity of the health policy issues under consideration and the need for sensitivity to the political decisions taken by the EU legislature with respect to how these issues should be tackled.^34^ This willingness to support the constitutionality of legislation that pursues the Union’s health policy objectives can also be observed in case law relating to food supplements^35^ and food flavourings.^36^ Therefore, based on the CJEU’s willingness to accept Article 114 as a legal basis for health integration, there is reason for optimism that the CJEU would support a Health Union built on the same constitutional foundations – provided, of course, that the Treaties have not already been amended to provide more bespoke competences.

## V. Institutional and agency powers

A key part of building a Banking Union was the creation of new powers for the ECB and the creation of powerful new European agencies such as the ESMA. This encroached significantly upon existing national regulatory competences, so it was predictable that several Member States resisted these reforms. The CJEU and the General Court have largely responded in a manner that has protected and reinforced the powers of the European banking institutions, even sometimes at the expense of robust and objective legal analysis.

There have been many cases concerning the powers of the ECB, but a good example that demonstrates the lengths to which the European courts will go to support the development of the Banking Union is provided in *Landeskreditbank*, in which the initial ruling of the General Court^37^ was upheld by the CJEU.^38^ It concerned the allocation of powers between the ECB and national central banks, specifically the question of which institution had the authority under the Single Supervisory Mechanism (SSM) Regulation to classify banks as “significant” or “less significant”, a decision that determined whether a bank was ultimately supervised by European or national authorities. The ECB refused to reclassify Landeskreditbank as “less significant”, thus allowing it to be supervised by the German national bank regulator, and Landeskreditbank challenged this refusal. The General Court, and then the CJEU, decided that the ECB’s decision was legitimate, and in the process held that the national banking authorities were only given powers by the SSM Regulation to support the work of the ECB, and that any autonomous rights to bank supervision previously enjoyed by national regulators had been transferred to the ECB. The robustness of the European courts’ reasoning in this case has been criticised, amongst other things on the basis that it went further than was necessary to decide the actual legal issues presented to it.^39^ However, this might have been due to the courts’ clear primary intent to protect the powers allocated to the European banking institutions, even if this had to come at the expense of solid legal reasoning.^40^

Again, there are reasons to be cautiously optimistic that the CJEU would protect the allocation of powers to EU health agencies in the same manner. The literature suggests that the CJEU tends not to be amenable to reviewing the scientific decisions of the European Food Safety Authority (EFSA), although it will review administrative decisions that the EFSA takes.^41^ The CJEU also recently confirmed that decisions of the European Medicines Agency (EMA) to publish clinical trial data were legitimate, thus supporting the EMA’s flagship policy of promoting transparency in relation to public access to clinical information.^42^ There are therefore broad indications that the CJEU is willing to support the EU’s health agencies in the discharge of their duties, support that would need to continue for a Health Union to operate effectively.

## VI. Conclusion

De Witte and Beukers observed that the CJEU’s decision in *Pringle* represented “a good mixture of legal principle and political pragmatism”.^43^ This largely sums up the CJEU’s approach in most of its subsequent case law on the Banking Union, and will equally need to be the guiding principle for how it responds to a future Health Union. This contribution has not sought to conduct a comprehensive review of the CJEU’s case law on banking or health, but has sought to present key ways in which the CJEU reacted to the establishment of a Banking Union in the aftermath of a destructive transnational financial crisis and then highlight evidence that might provide an indication of how the CJEU would react to the establishment of a Health Union in the aftermath of the similarly destructive transnational health crisis resulting from the emergence of the COVID-19 virus. The objective has been to illustrate how we can learn lessons from the financial crisis about the resilience and flexibility of existing EU legal structures as we seek long-term legislative and institutional solutions to the challenges created by the COVID-19 pandemic. The tentative conclusion to be drawn from this exploration of CJEU case law is that there are reasons to be optimistic that the CJEU would constructively support the development of a Health Union and that we can identify political and policy-orientated lines of argument based on those used in parallel situations in the banking case law that might help to secure such support.

## Footnotes

1 See special issue 15(3) of *Comparative European Politics* on the Rise of a European Healthcare Union, and the introductory entry: H Vollaard and D Sindbjerg Martinsen, “The rise of a European healthcare union” (2017) 15(3) Comparative European Politics 337.

2 C Luena, “Time for a European Health Union” (*theparliamentmagazine.eu*, 5 May 2020) <https://www.theparliamentmagazine.eu/news/article/time-for-a-european-health-union>.

3 Regulation 1024/2013, OJ L 287, 29.10.2013, pp 63–89.

4 Regulation 806/2014, OJ L 225, 30.7.2014, pp 1–90.

5 These three aspects were identified following a reading of the literature on the CJEU and the Banking Union, in particular M Chiti, “The European Banking Union in the Case Law of the Court of Justice of the European Union” in M Chiti and V Santoro (eds), *The Palgrave Handbook of European Banking Union Law* (London, Palgrave Macmillan 2019) pp 105–34.

6 C-370/12 *Pringle* [2012] ECLI:EU:C:2012:756.

7
ibid, para 30.

8
ibid, paras 35, 80.

9 B de Witte and T Beukers, “The Court of Justice approves the creation of the European Stability Mechanism outside the EU legal order: *Pringle*” (2013) 50 Common Market Law Review 805, 826.

10

N Moloney, “European Banking Union: Assessing its Risks and Resilience” (2014) 51 Common Market Law Review 1609, 1624.

11 G Beck, “The Court of Justice, the Bundesverfassungsgericht and Legal Reasoning during the Euro Crisis: The Rule of Law as a Fair-Weather Phenomenon” (2014) 20(3) European Public Law 539.

12 Indeed, given the necessity of deeper cooperation on banking to ensure that the financial crisis was controlled and not repeated, the decision has been described as not only essential, but also unsurprising: A Hinarejos, “The Court of Justice of the EU and the Legality of the European Stability Mechanism” (2013) 72(2) Cambridge Law Journal 237.

13 C-202/18 *Rimšēvičs v Latvia* [2019] ECLI:EU:C:2019:139.

14 Contained in Protocol No 4, annexed to the Treaty on European Union and the Treaty on the Functioning of the European Union, OJ C 83, 30.3.2010, p 230.

15 R Smits, “A National Measure Annulled by the European Court of Justice, or: High-level Judicial Protection for Independent Central Bankers” (2020) 16 European Constitutional Law Review 120.

16 D Sindbjerg Martinsen, “Towards an Internal Health Market with the European Court” (2005) 28(5) West European Politics 1035.

17 C-286/82 *Luisi and Carbone v Ministero dello Tesoro* [1984] ECLI:EU:C:1984:35.

18 C-158/96 *Kohll v Union des caisses de maladie* [1998] ECLI:EU:C:1998:171.

19 A Kaczorowska, “A Review of the Creation by the European Court of Justice of the Right to Effective and Speedy Medical Treatment and its Outcomes” (2006) 12(3) European Law Journal 345.

20 C-159/90 *Society for the Protection of Unborn Children Ireland v Grogan and Others* [1991] ECLI:EU:C:1991:378.

21 C-405/98 *Gourmet International Products* [2001] ECLI:EU:C:2001:135.

22 Case T-177/13 *Test Bio Tech* [2106] ECLI:EU:T:2016:736.

23 Case T-204/11 *Spain v Commission* [2015] ECLI:EU:T:2015:91.

24 In the *ESMA* case considered below, the CJEU diverged significantly from the Advocate General on the interpretation of the scope of Art 114 TFEU.

25 Case C-491/01 British American Tobacco (Investments) and Imperial Tobacco [2002] ECLI:EU:C:2002:741, paras 203–71.

26 Case C-360/11 *Commission v Spain* [2013] ECLI:EU:C:2013:17, para 66.

27 C-270/12 *United Kingdom v Parliament and Council* [2014] ECLI:EU:C:2014:18.

28 H Marosola, “Bridging the constitutional gap in EU executive rule-making: the court of justice approves legislative conferral of intervention powers to European securities markets authority” (2014) 10 European Constitutional Law Review 500.

29 E Howell, “The European Court of Justice: selling us short?” (2014) 11(3) European Company and Financial Law Review 45.

30
ibid.

31 E Ferran, “European Banking Union: Imperfect, But It Can Work” (2014) University of Cambridge Faculty of Law Research Paper No. 30/2014, 18.

32 C-376/98 *Germany v Parliament and Council (Tobacco Advertising 1)* [2000] ECLI:EU:C:2000:544; C-380/03 *Germany v Parliament and Council (Tobacco Advertising 2)* [2006] ECLI:EU:C:2006:772.

33 C-547/14 *Philip Morris Brands* [2016] ECLI:EU:C:2016:325; C-477/14 *Pillbox 38* [2016] ECLI:EU:C:2016:324; C-358/14 *Poland v Parliament and Council* [2016] ECLI:EU:C:2016:323; C-220/17 *Planta Tabak* [2019] ECLI:EU:C:2019:76.

34 The Union’s margin of discretion to use the powers in Art 114 to regulate tobacco products is repeatedly emphasised by the CJEU, such as in *Philip Morris Brands*, at para 166: “[T]he EU legislature must be allowed broad discretion in an area such as that involved in the main proceedings, which entails political, economic and social choices on its part, and in which it is called upon to undertake complex assessments”.

35 Joined Cases C-154/04 and C-155/04 *Alliance for Natural Health* [2005] ECLI:EU:C:2005:449.

36 C-66/04 *United Kingdom v Parliament and Council* [2005] ECLI:EU:C:2005:743.

37 Case T-122/15 *Landeskreditbank Baden-Württemberg – Förderbank v European Central Bank* [2017] ECLI:EU:T:2017:337.

38 Case C-450/17 P *Landeskreditbank Baden-Württemberg – Förderbank v European Central Bank* [2019] ECLI:EU:C:2019:372.

39 P Schammo, “Matching or clashing? *Landeskreditbank Baden-Württemberg v ECB* and the decision of the German Bundesverfassungsgericht on the Banking Union” (28 November 2019) <https://ssrn.com/abstract=3495226 or https://doi.org/10.2139/ssrn.3495226>.

40 F Annunziata, “European Banking Supervision in the Age of the ECB: *Landeskreditbank Baden-Württemberg – Förderbank v. ECB*” (2020) European Business Organization Law Review 10.1007/s40804-019-00170-y.

41 A Alemanno and S Mahieu, “The European Food Safety Authority before European Courts” (2008) 3(5) European Food and Feed Law Review 320; S Gabbi et al, “Controlling the European Food Safety Authority” in M Scholten and A Brenninkmeijer (eds)*, Controlling EU Agencies: The Rule of Law in a Multi-jurisdictional Legal Order* (Cheltenham, Edward Elgar Publishing 2020) pp 196–214.

42 Case C-175/18 P *PTC Therapeutics International v EMA* [2020] ECLI:EU:C:2020:23; Case C-178/18 P *MSD Animal Health Innovation and Intervet international v EMA* [2020] ECLI:EU:C:2020:24.

43 B de Witte and T Beukers, “The Court of Justice approves the creation of the European Stability Mechanism outside the EU legal order: *Pringle*” (2013) 50 Common Market Law Review 805, 848.

